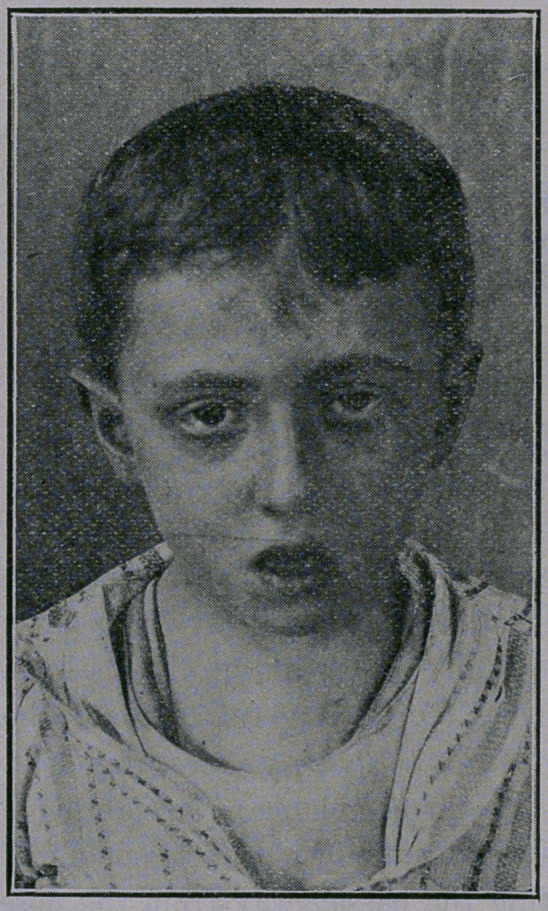# Obstructed Nasal Breathing in Infancy and Childhood

**Published:** 1912-08

**Authors:** E. Mather Sill

**Affiliations:** New York City. Attending Physician in Diseases of Children at Good Samaritan Dispensary; Lecturer in Diseases of Children at the New York Polyclinic Hospital and Medical School


### TEXAS MEDICAL JOURNAL.

Established July, 1885

F. E. DANIEL, M. D., - - - - - Editor, Publisher and Proprietor

Published Monthly.—Subscription, $1.00 a Year.

VOL. XXVIII. AUSTIN, AUGUST, 1912. No. 2.

The publisher is not responsible for the views of the contributors.

### Original Articles.

For Texas Medical Journal.

### Obstructed Nasal Breathing in Infancy and Childhood.—Is There Developing Among us a Modern Race of Mouth Breathers?

BY E. MATHER SILL, M. D., NEW YORK CITY.

Attending Physician in Diseases of Children at Good Samaritan Dispensary; Lecturer in Diseases of Children at the New York Polyclinic Hospital and Medical School.

In connection with medical inspection of the schools, hospitals, child welfare exhibits, and from the child specialist we hear a great deal about "adenoids." To many the meaning of this term is already familiar, but for the benefit of that great number who do not yet understand it I will endeavor to make its meaning clear. It comes from the Greek (*aden-eidos,* resemblance), resembling a gland, and that is what adenoids are, an abnormal enlargement of normal glandular tissue, obstructing the passageway from the nose to the throat, and so making breathing through the mouth necessary.

This condition has no doubt existed for ages, but has only within comparatively recent years been brought prominently before the general medical practitioners and the public.

Parents, and even some physicians, hardly appreciate what a serious state of things adenoids may lead to, both in infancy and childhood. Too much emphasis can not be placed on the fact that adenoid growths are responsible for more minor troubles in infants and childhood than any other abnormal condition. The adenoid tissue in children is very susceptible to changes in its blood supply, from slight causes, such as repeated colds, which keep it continuously congested, and so even a small amount of adenoid tissue in a small child, whose nasal passages are small, may become as formidable as a greater amount in a larger child. More than three-fourths of all the cases of adenoids occur between the ages of one and fifteen years; indeed, many cases develop even before the end of the first year, and these last are very apt to be overlooked or neglected until serious and permanent damage has been done.

Babies so affected will often have a watery discharge from the nose, which reddens and inflames the nostrils and irritates the skin of the lips. The mouth may be kept open, the breathing is loud, a hoarse, dry cough may be present, or the voice may have a nasal twang. Nursing babies severely affected are only able to take the breast for a short time, without dropping the nipple in order to breathe. Such babies are subject to frequent colds in the head, and in fact are rarely free from snuffles. Peculiar crowing sounds are sometimes complained of, or a clicking may be made when the child nurses.

When children two or three years of age or older are suffering from adenoids they have more or less constant cold in the head, with catarrh, a persistent, dry, hacking cough, which is usually worse at night or in the early morning; they keep their mouths open most of the time, which gives then a vacant or stupid expression; their breathing at night is loud and frequently accompanied by snoring. Such children are poor sleepers and apt to be restless at night.

Children with adenoids of long standing are frequently pale, malnourished, fretful, with bad tempers, narrow, sunkened chests, drooping eyelids, dull, listless expressions, contracted nasal passages, with prominent veins at the root of the nose. This condition ought to be easily recognized, but to expect the whole classical train of symptoms to appear would result in allowing many little sufferers in whom the disease is not far advanced to remain without treatment.

When children, especially infants, have not this characteristic appearance, adenoids may be inferred when there is a history of obstructed breathing through the nose, evidenced by snoring, frequent colds in the head during the winter, inability to nurse long, and attacks of earache. This condition in the child the examination of a physician will usually confirm as being caused by adenoids.

Children with adenoids frequently have a coated tongue, as a result of indigestion, due to swallowing the catarrhal secretion, which is always present. The tonsils may be enlarged above normal, and children with enlarged tonsils always have adenoids, but adenoids are frequent where there is no enlargement of the tonsils.

Children with adenoids are particularly liable to earache and headache, and not infrequently have abscess in the middle ear. It has been stated by numerous authors that over 90 per cent of all adenoid cases are accompanied by some degree of deafness, and it is even now generally conceded that adenoids are the principal cause of deaf mutism. Some of the worse nasal deformities result from this condition, according to best authorities. Defective speech (nasal voice), anemia, listlessness, frequent attacks of indisposition and nervousness are also common with this trouble.

Children with adenoids are usually dull and inattentive in school and subject to fits of bad temper. The physician is often consulted for earache, persistent cold in the head, cough, anemia, poor appetite, malnutrition, and upon examination the real underlying cause is found to be adenoid growth. Here we have a serious state of things if not attended to but a condition easily amenable to proper treatment, with immediate and gratifying results.

What is the best and only satisfactory method of dealing with adenoids in childhood? Treatment without operation is of little or no avail, as I have seen in a great many cases. Why then wait with the vain hope that the adenoid growth may atrophy, or the child "outgrow" it? Why waste time and subject the poor little patients to the grave results that are sure to follow? Why not remove the growth at once, when it is so quickly and easily done by one experienced, with little or no danger and with immediate good results? If adenoids are left they very rarely atrophy, and then only partially after puberty.

What is the result if adenoids are not removed? A stunted growth, a narrow, sunken chest, or "chicken breast," frequent, severe colds, more or less continuous catarrh, sore throat, impaired mentality, with dullness and stupidity, frequent ear troubles and impaired hearing, a nasal voice, changed facial appearance, with sometimes idiotic expression, great susceptibility to measles, scarlet fever, diphtheria, pneumonia, asthma, hay fever, and frequently enlargement of the glands of the neck, since adenoids are the cause of this last affection in fully three-quarters of the cases. Remove the adenoids, and the glands disappear.

Adenoids and chronically enlarged tonsils are a common gateway for the entrance of turberculous and various other germs into the system; they may also be the cause of many reflex troubles, such as cough, bed-wetting, and a great variety of minor difficulties.

It hardly seems possible that any sane parent who has a child with adenoids or one whose symptoms indicate their existence could hesitate about the proper course to pursue, and yet, far too frequently, I regret to say, parents delay consulting a specialist and having such immediate treatment as would bring health and happiness to the little sufferer.

None but one who has witnessed the beneficial results obtained from operation on thousands of little patients can realize or appreciate the wonderful change for the better that may be brought about by means of this radical treatment.

## Figures and Tables

**Figure f1:**